# Scoliosis: Congenital, Neuromuscular, Syndromic, and Other Nonidiopathic Types, Understanding and managing the condition: A practical guide for familiesBy Tenner J. Guillaume, Walter H. Truong, Danielle Harding, Lily Collison, and Cheryl Tveit Gillette Children's Healthcare Series. St Paul, MN: Gillette Children's Healthcare Press, 2025, £45.00 (paperback), £10.00 (eBook), pp. 316, ISBN: 9781952181214

**DOI:** 10.1111/dmcn.70054

**Published:** 2025-10-31

**Authors:** Brian Snyder

**Affiliations:** ^1^ Boston Children's Hospital – Orthopedic Surgery Boston MA USA

Part of the Gillette Children's Healthcare Book Series, this new title from Gillette Children's Healthcare Press and Mac Keith Press provides a lucid and comprehensive review of the etiology, pathoanatomy, and treatment of non‐idiopathic types of spinal deformity. Based on evidence‐based literature, it is written in a style that is accessible to patients, their families and caregivers, as well as being appropriate for healthcare professionals, researchers, educators, and students. The focus is on the four main types of non‐idiopathic spine deformity. (1) Congenital – errors in vertebral development (i.e. failures of formation and/or segmentation). (2) Neuromuscular – consequence of neuromuscular conditions affecting the nervous and/or muscular systems (e.g. cerebral palsy, spinal muscular atrophy, spina bifida, Duchenne muscular dystrophy, arthrogryposis multiplex congenita). (3) Syndromic – induced by a genetic syndrome (e.g. Rett syndrome, autism spectrum disorder, neurofibromatosis, Down syndrome, Ehlers‐Danlos syndrome, Prader‐Willi syndrome). (4) Other non‐idiopathic – provoked by spinal cord malformations – syrinx, Chiari malformation type I, or tethered cord syndrome.

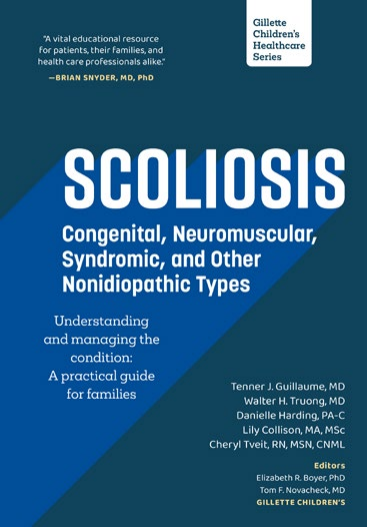

The authors introduce the three‐dimensional nature of spine pathoanatomy, where the deformity occurs in all three anatomic planes involving different functional and structural regions of the spine (cervical, thoracic, lumbar, and sacrum/pelvis). Scoliosis is the projection of the deformity onto the coronal plane, kyphosis/lordosis is the projection of the deformity onto the sagittal plane, and torsion/rotation is the projection of the deformity onto the axial plane. Spine development is examined from in utero to skeletal maturity, highlighting how the pubertal growth spurt accelerates deformity progression in adolescence, and also considers issues of adults with distorted spines during aging. The authors explain how spine deformity is classified according to the cause (congenital, neuromuscular, syndromic, or non‐idiopathic spinal cord malformations) and age at onset (early – infantile or juvenile and later – adolescent).

Common signs and symptoms of spine pathoanatomy that parents and care providers should notice are outlined and the importance of timely referral to a spine specialist is emphasized. Creating an effective treatment plan is predicated on a comprehensive medical history, physical exam, and imaging studies that includes multi‐planar spine radiographs with or without magnetic resonance imaging to visualize all relevant musculoskeletal pathology. For each category of spine deformity, the book is structured to guide readers through the various treatment modalities, the selection based on the underlying diagnosis, patient age, anatomic location, severity of deformity, and coexisting medical comorbidities.

Chapters 6 through 9 are dedicated to an overview of therapies, covering both nonsurgical options (observation, bracing, casting, physical therapy, alternative treatments) and surgical interventions (spinal fusion, growth‐friendly spine instrumentation, halo gravity traction). Chapter 9 delves into specific considerations relevant to congenital, neuromuscular, syndromic, or non‐idiopathic cord abnormalities. A unique aspect of this book is the inclusion of the lived experiences of children with scoliosis and their families. Complementing the didactic medical information, personal stories are interspersed throughout the text, offering a human dimension to this disorder.

*Scoliosis: Congenital, Neuromuscular, Syndromic, and Other Nonidiopathic Types* serves as a vital educational resource for patients, their families, and healthcare professionals alike, emphasizing evidence‐based best practices and providing guidance for exploring the literature and further research. Beyond the pragmatic medical information provided, the text underscores the critical partnerships among patients, their families/caretakers, and healthcare professionals in optimizing outcomes for individuals living with this lifelong condition.

## Data Availability

Not required.

